# Altered Brain Structural Reorganization and Hierarchical Integrated Processing in Obesity

**DOI:** 10.3389/fnins.2022.796792

**Published:** 2022-03-18

**Authors:** Peng Zhang, Guo-wei Wu, Li-rong Tang, Feng-xia Yu, Meng-yi Li, Zheng Wang, Zheng-han Yang, Zhong-tao Zhang, Han Lv, Yang Liu, Zhen-chang Wang

**Affiliations:** ^1^Department of Radiology, Beijing Friendship Hospital, Capital Medical University, Beijing, China; ^2^Chinese Institute for Brain Research, Beijing, China; ^3^Department of Clinical Psychology Center, Beijing Anding Hospital, Capital Medical University and National Clinical Research Center for Mental Disorders and Beijing Key Laboratory of Mental Disorders, Beijing, China; ^4^Medical Imaging Center, Beijing Tongren Hospital, Capital Medical University, Beijing, China; ^5^Department of General Surgery, Beijing Friendship Hospital, Capital Medical University and National Clinical Research Center for Digestive Diseases, Beijing, China

**Keywords:** obesity, imaging, neuroscience, eating behaviors, magnetic resonance

## Abstract

The brain receives sensory information about food, evaluates its desirability and value, and responds with approach or withdrawal. The evaluation process of food in the brain with obesity may involve a variety of neurocircuit abnormalities in the integration of internal and external information processing. There is a lack of consistency of the results extant reported for aberrant changes in the brain with obesity that prohibits key brain alterations to be identified. Moreover, most studies focus on the observation of neural plasticity of function or structure, and the evidence for functional and structural correlations in the neuronal plasticity process of obesity is still insufficient. The aims of this article are to explore the key neural structural regions and the hierarchical activity pattern of key structural nodes and evaluate the correlation between changes in functional modulation and eating behavior. Forty-two participants with obesity and 33 normal-weight volunteers were recruited. Gray matter volume (GMV) and Granger causality analysis (GCA) were performed using the DPARSF, CAT12, and DynamicBC toolbox. Compared with the normal weight group, the obesity group exhibited significantly increased GMV in the left parahippocampal gyrus (PG). The obesity group showed decreased causal inflow to the left PG from the left orbitofrontal cortex (OFC), right calcarine, and bilateral supplementary motor area (SMA). Decreased causal outflow to the left OFC, right precuneus, and right SMA from the left PG, as well as increased causal outflow to the left middle occipital gyrus (MOG) were observed in the obesity group. Negative correlations were found between DEBQ-External scores and causal outflow from the left PG to the left OFC, and DEBQ-Restraint scores and causal inflow from the left OFC to the left PG in the obesity group. Positive correlation was found between DEBQ-External scores and causal outflow from the left PG to the left MOG. These results show that the increased GMV in the PG may play an important role in obesity, which may be related to devalued reward system, altered behavioral inhibition, and the disengagement of attentional and visual function for external signals. These findings have important implications for understanding neural mechanisms in obesity and developing individual-tailored strategies for obesity prevention.

## Introduction

Obesity is defined as a disproportionate body weight for height with an excessive accumulation of adipose tissue. According to the World Health Organization (WHO), more than 650 million adults were obese in 2016 (World Health Organization [WHO], 2021). Obesity causes a wide range of diseases, such as cardiovascular diseases, gastrointestinal disorders, joint and muscular disorders, and respiratory problems, and some of these comorbidities are considered as an important risk factor for increasing mortality of diseases (Williams et al., 2015). Although obesity is now mainly considered an imbalance in energy intake and expenditure, which is caused by neurobehavioral disorder, the underlying mechanisms of this neurobehavioral disorder remain largely unelucidated.

The brain receives sensory information about food, evaluates its desirability and value, and responds with approach or withdrawal. Growing evidence suggests that the evaluation process of food in the brain with obesity may involve a variety of neurocircuit abnormalities in the integration of internal and external information processing (Volkow et al., 2011). With the deepening of obesity neuroimaging research, many regional functional brain regions and networks have been identified to be involved in the neural process of obesity (Kenny, 2011; Hendrikse et al., 2015; Ziauddeen et al., 2015; Steward et al., 2019).

At the functional magnetic resonance imaging (fMRI) level, a series research results and our previous results have shown that multiple brain regions involved in reward, sensorimotor, emotional, attentional, behavioral control, and cognitive functional areas were found participating in the neural processing of obesity (Kenny, 2011; Hendrikse et al., 2015; Doucet et al., 2018; Steward et al., 2019; Zhang et al., 2020). Studies have found that the caudate and putamen have been implicated in abnormal eating behavior and associated with high body mass index (BMI) and deficiencies in reward processing (Stice et al., 2008; Zhang et al., 2020). There is evidence that BMI is negatively correlated with activation throughout diverse loci in the frontal inhibitory regions, including superior frontal gyrus, middle frontal gyrus, ventrolateral prefrontal cortex (PFC), medial PFC, and orbitofrontal cortex (OFC), when subjects are required to inhibit prepotent responses to appetizing foods (Batterink et al., 2010). Some scholars observed that increased BMI was associated with diminished lateral PFC responses during food attentional bias, which may reflect decreased goal-directed control of food choices following outcome devaluation (Janssen et al., 2017). Moreover, previous studies have further shown that there is abnormal functional connectivity between key functional brain regions in the brain with obesity (Donofry et al., 2020; Zhang et al., 2020; Syan et al., 2021). Furthermore, our previous studies, based on the theory of the hierarchical effect, also have shown directional and hierarchical signal processing between key reward regions and multiple functional areas (Zhang et al., 2019, 2021). In addition to the research on fMRI, the research on structural magnetic resonance imaging (sMRI) of neural microstructure has also made progress. Meta-analyses of structural imaging studies of obesity have shown that greater BMI is associated with decreased gray matter volume (GMV) in the OFC (Chen et al., 2020). The results of another meta-analysis have suggested that the BMI was associated with lower GMV in areas including the medial PFC, bilateral cerebellum, and left temporal pole (García-García et al., 2019). The findings of studies have shown that GMV reductions of the bilateral inferior frontal gyrus, left middle temporal cortex, left precentral gyrus, and cerebellum and increased GMV of the left cuneus, left middle frontal gyrus, and left inferior occipital gyrus were observed in obesity (Herrmann et al., 2019).

Although functional and structural magnetic resonance imaging studies have identified some aberrant changes in the brain with obesity, there is a lack of consistency of the results reported that prohibits key brain alterations to be identified. Moreover, most studies focus on the observation of neural plasticity of function or structure, and the evidence for functional and structural correlations in the neuronal plasticity process and hierarchical functional activity pattern of obesity is still insufficient. Therefore, in this study, we aimed to evaluate the key neural structural regions by voxel-based morphometry (VBM), a classical sMRI method, in obese adults. Moreover, Granger causality analysis (GCA), a data-driven technique of fMRI reflecting the directed functional (“causal”) interaction characterization of brain functional circuits, was conducted to investigate the hierarchical activity pattern of key structural nodes. We hypothesized that adults with obesity would have one or more key areas of gray matter structural change compared with controls, and that these key structural areas might have specific hierarchical integrated processing pattern with other regions of the whole brain that might be associated with abnormal eating behavior.

## Materials and Methods

### Participants

Forty-two participants with obesity (BMI > 30.0 kg/m^2^) and 33 normal-weight volunteers (18.5 kg/m^2^ < BMI < 25.0 kg/m^2^) of similar ages (18–55) were recruited in this study. All selected participants fit the following inclusion criteria for the present study: righthanded; non-smokers; stable body weight (<5% reported change during the previous 3 months); no history of metabolic, neurological, or psychiatric disease [as assessed with the General Health Questionnaire (Lee et al., 2006)]; no drug dependence; no MRI contraindications (metal implants or claustrophobia); and no organ dysfunction or taking any current medication that could affect the central nervous system. Individuals who had a waist circumference > interior diameter of the MRI scanner were excluded.

Participants underwent MRI scan after an overnight fast of at least 10 h. To minimize circadian influences, participants’ scans were scheduled between 6 and 8 a.m. To keep participants in the waking state during the scan, participants were instructed regarding the date of examination 1–2 weeks before the scan to allow them to adjust their circadian rhythm. Immediately before each scan, participants were asked to answer the following question: “How hungry do you feel?” by marking a vertical line on a 100-mm visual analog scale anchored with ends labeled “I am not hungry at all (0)” and “I have never been more hungry (100).” Prior to scanning, the Dutch Eating Behavior Questionnaire (DEBQ) (van Strien et al., 1986) was used to assess participants’ eating behavior. It consisted of three subscales reflecting emotional (psychosomatic) eating (eating in response to emotional distress), external eating (eating in response to external food cues, such as food sight and smell), and restraint eating (overeating in the wake of attempted restraint). DEBQ has 33 items in total, and each item was measured on a five-point Likert scale. The previous study demonstrated that the DEBQ has good reliability and validity in assessing eating behaviors in the Chinese population (Wu et al., 2017). During the scan, which lasted approximately 12 min, tight but comfortable foam padding and earplugs were used to minimize head motion and reduce imaging noise, respectively.

### Image Acquisition

All images were obtained using a 3-T Discovery MR750w scanner (General Electric, Milwaukee, WI, United States) with an eight-channel phased array coil. To exclude any possible abnormality in the brain, a conventional brain axial T2 sequence first was scanned. High-resolution three-dimensional (3D) structural T1-weighted images were acquired using a 3D BRAVO sequence with the following parameters: 196 slices with 1-mm thickness; repetition time (TR)/echo time (TE) = 8.8/3.5 ms; inversion time (TI) = 450 ms; field of view (FOV) = 24 × 24 cm; matrix = 256 × 256; and flip angle = 15°, resulting in an isotropic voxel size of 1 × 1 × 1 mm^3^. Functional images were obtained using a multislice gradient-echo echo-planar imaging (EPI) sequence with the following imaging parameters: 28 slices with 4-mm slice thickness and a 1-mm gap; 200 time points; TR = 2,000 ms; TE = 35 ms; flip angle = 90°; FOV = 240 × 240-mm axial slices providing whole-brain coverage.

### Image Analysis

#### Structural Data Processing and Voxel-Based Morphometry Analysis

The CAT12 package^1^ implemented in the Statistical Parametric Mapping (SPM) software^2^ was used for post-processing structural data. SPM12 was installed in MATLAB 2016a (Math Works, Natick, MA, United States). First, the images were manually reoriented to place the anterior commissure at the origin and the anterior–posterior commissure in the horizontal plane if necessary. Then the structural images acquired by the 3D-BRAVO sequence were segmented into gray matter (GM), white matter (WM), and cerebrospinal fluid (CSF) areas using the unified standard segmentation model in SPM12. The individual GM and WM components were normalized to the standard Montreal Neurological Institute (MNI) space using the Diffeomorphic Anatomical Registration through Exponentiated Lie Algebra (DARTEL) algorithm (Ashburner, 2007) after segmentation. The normalized GM and WM components were modulated to generate the relative GMV and WM volume (WMV) by multiplying by the non-linear part of the deformation field at the DARTEL step. The GMV/WMV images were spatially smoothed with a 6-mm FWHM isotropic Gaussian kernel.

#### Functional Data Processing and Granger Causality Analysis

Pre-processing of the functional images was conducted using the Data Processing and Analysis of Brain Imaging (DPABI^3^) and GRETNA^4^ toolkits. Most of the toolkit functions are based on SPM12. The pre-processing main steps were as follows: the first 10 time points were removed for signal equilibrium and the participants’ adaptation to the scanning noise. Then slice timing, head motion correction, and spatial normalization to the SPM12 MNI template were performed. A sample-specific DARTEL (Ashburner, 2007) template was created using structural images from all participants. The EPI volumes were then normalized to the MNI space using the DARTEL template and the corresponding flow field, and resampled to a voxel size of 3 × 3 × 3 mm. An isotropic Gaussian kernel (full-width-at-half maximum = 6 mm^3^) was used to spatially smooth the images. For the purpose of an effective connectivity (EC) analysis, additional detrending was performed and white matter signals, CSF, global brain signals, six standard head motion parameters, the derivative of the standard motion parameters to account for a one-frame delay in the effect of motion on the blood oxygenation level-dependent (BOLD) signal, and the 12 corresponding squared items were regressed out as nuisance covariates (Power et al., 2014). MRI time points that were severely affected by motion were removed using a “scrubbing method” [framewise displacement (FD) value > 0.5 mm and ΔBOLD of DVARS > 0.5%], and <5% of time points were scrubbed per subject. Finally, band-pass temporal filtering (0.01–0.08 Hz) was applied to reduce low-frequency drifts and high-frequency physiological noise.

Granger causality analysis was used to describe the EC between the reference time series of the seed regions and the time series of each voxel within the whole brain. The voxel-wise GCA was performed using the DynamicBC toolbox (Dynamic Brain Connectome^5^). Granger causality estimates the causal effect of the seed region on every other voxel in the brain (*X* to *Y* effect), as well as the *Y* to *X* effect, the causal effect of every voxel in the brain (*Y*) on the seed region (*X*). Granger causality is often used for fMRI data analysis *via* vector autoregression as follows:


(1) $$Y_{t}=\sum_{k=1}^{p}A_{k}\,X_{\left(t-k\right)}+\sum_{k=1}^{p}B_{k}\,Y_{\left(t-k\right)}+C\,Z_{t}+E_{t}$$

(2) $$X_{t}=\sum_{k=1}^{p}A_{k}^{\prime }\,Y_{\left(t-k\right)}+\sum_{k=1}^{p}B_{k}^{\prime }\,X_{\left(t-k\right)}+C^{\prime }\,Z_{t}+E_{t}^{\prime }$$

where *X_t* and *Y_t* represent two time series, *A_k* and $A_{k}^{\prime }$ are signed-path coefficients, *B_k* and $B_{k}^{\prime }$ are autoregression coefficients, *E_t* and $E_{t}^{\prime }$ are residuals, and *Z_t* represents covariates (e.g., head motion, global trend, and time series from certain brain areas). The time series *X_t* significantly causes the time series *Y_t* if the signed-path coefficient *A_k* is significantly larger (Zang et al., 2012). Likewise, *Y_t* can be defined as a significant Granger cause to *X_t* if the signed-path coefficient $A_{k}^{\prime }$ is significantly larger. In this study, the time series of the regions from VBM results was defined as the seed time series *X*, and the time course of voxels within the whole brain was defined as *Y*. The bivariate coefficient GCA was performed to explore the Granger causal influence between the seed region of interest (ROI) and each voxel of the whole brain. Finally, the GCA maps for all participants were converted to *z*-values using Fisher’s *r*-to*-z* transformation to improve the normality.

### Quality Control

The participants were asked to close their eyes, stay awake, breathe evenly, and try to avoid specific thoughts. Directly after the scan, they were asked whether they had fallen asleep, and none of the participants reported that they had. All images were manually reviewed for data quality. Images with the most severe artifact, irregularities, and/or poor image quality were rejected and excluded from processing and analysis. Any participants with more than 2-mm displacement or 2° of head rotation were excluded in a head motion correction step. Additionally, FD was calculated, which considers measures of voxelwise differences in motion in its derivation as a measure of the microhead motion of each participant. Finally, the normalized GMV maps were used as nuisance covariates to control possible GMV influences on the functional analysis results.

### Statistical Analyses

The chi-squared test was used to analyze gender distribution, and independent two-sample *t*-tests were used to assess differences between the obese group and the normal-weight group in clinical data. The above analyses were conducted with SPSS version 20.0 (SPSS Inc., Chicago, IL, United States), with the significance threshold set at *p* < 0.05.

For T1 3D BRAVO images, using the general linear model (GLM) in SPM12, independent two-sample *t*-tests were applied to compare the GMV and WMV differences in the whole brain between the obesity group and the normal-weight group [family wise error (FWE) correction with *p* < 0.05], and age, gender, education, mean FD, and total intracranial volume served as nuisance covariates. For functional images, within each group, the mean *z*-values of the seed time series to the time course of voxels within the whole brain and the time course of voxels within the whole brain to seed time series maps were calculated. Two-sample *t*-tests were performed to determine differences in the ECs of key regions between the two groups, with the age, gender, education, mean FD, and GMV as covariates. Statistical significance was set at *p* < 0.05 and corrected for multiple comparisons using the false discovery rate (FDR). Pearson’s correlation analysis was performed to explore any potential associations between the participants’ clinical data (such as the DEBQ scores and VAS scores) and the brain changes (GMV and ECs) after removing any age, gender, and education effects. Kolmogorov–Smirnov test was conducted to assess the normality of all the data in this study.

## Results

### Demographics and Clinical Data

Four participants with obesity and three normal-weight volunteers were excluded due to head movement or poor image quality. Six participants with obesity were excluded due to metabolic disease. Therefore, the sample of this study consisted of 32 individuals with obesity and age-, gender-, education-, and handedness-matched 30 normal-weight volunteers. There were no differences in age, gender, education, hours of fasting, subjective degree of hunger, and DEBQ-External scores between the obesity group and normal-weight group. BMI, DEBQ-Emotional scores, and DEBQ-Restraint scores were higher for the obesity group than for the normal-weight group. The demographic and clinical characteristics are summarized in Table 1.

**TABLE 1:** Demographic and clinical characteristics of the participants.

	Participants with obesity (*n* = 32)	Participants with normal weight (*n* = 30)	*p*-value
Gender (male/female)	6/26	10/20	0.190^a^
Age (years)	28.06 ± 7.60	25.96 ± 3.81	0.180^b^
BMI	41.06 ± 5.52	21.37 ± 1.41	<0.0001*^b^
Education (years)	13.91 ± 2.94	14.60 ± 1.63	0.295^b^
Hours fasting (hours)	11.28 ± 1.28	11.20 ± 1.00	0.795^b^
Subjectively rated hunger (mm)	54.19 ± 18.21	49.92 ± 16.65	0.366^b^
DEBQ-Emotional	2.85 ± 0.91	2.37 ± 0.72	0.033*^b^
DEBQ-External	3.15 ± 0.49	2.89 ± 0.49	0.053^b^
DEBQ-Restraint	2.91 ± 0.76	2.23 ± 0.51	0.0001*^b^

*Data are presented as the mean ± standard deviation.*

*p < 0.05.

*^a^Chi-square test.*

*^b^Independent t-test.*

### Brain Structural Alterations

**FIGURE 1:**
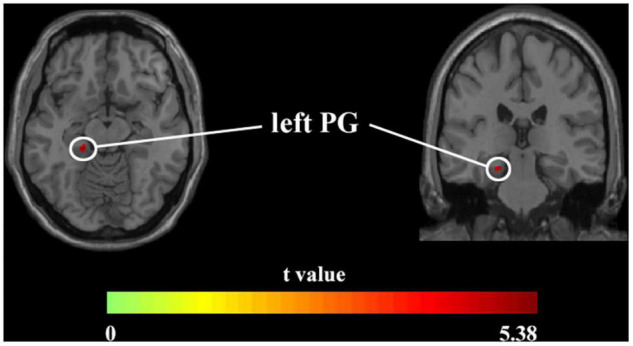
Axial and coronal images show that the obesity group exhibited increased GMV in the left PG compared with the normal-weight group (FWE-corrected *p* < 0.05). FWE, family-wise error; GMV, gray matter volume; PG, parahippocampal gyrus.

Voxel-based morphometry analysis identified the increased GMV of the obesity group in the left parahippocampal gyrus (PG) compared with the normal-weight group (*p* < 0.05, FWE corrected) (Figure 1 and Table 2). However, we did not find any significant differences in WMV between the two groups.

**TABLE 2:** Difference in gray matter volume between the obesity and normal-weight groups.

Region	MNI coordinates			Size (voxels)	Peak *t*-value
	
	x	y	z		
Left parahippocampal gyrus	−21	−31	−13	57	5.3821

*MNI, Montreal Neurological Institute.*

### Effective Connectivity Alterations of Key Structural Region

Based on the results of the GMV analysis, the left PGs were defined as the key seed regions to analyze the EC.

#### Effective Connectivity From the Whole Brain to Left Parahippocampal Gyrus

**FIGURE 2:**
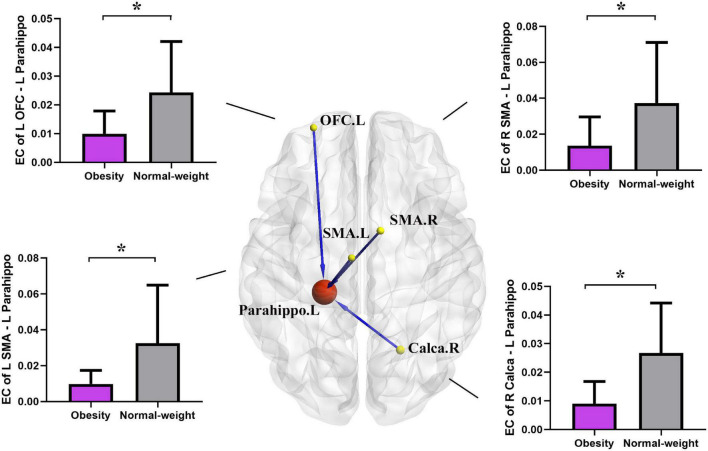
Altered effective connectivity (EC) in the obesity group compared with the normal-weight group (*p* < 0.05, FDR corrected). The red node indicates the key structural region (i.e., parahippocampal gyrus). The yellow nodes and blue lines indicate the input brain region (SMA, OFC, and Calca) and decreased EC input to the key region. Parahippo, parahippocampal gyrus; SMA, supplementary motor area; OFC, orbitofrontal cortex; Calca, calcarine; EC, effective connectivity; FDR, false discovery rate; GMV, gray matter volume; L, left; R, right. The size of the sphere represents the voxel size of the brain region based on the results of the GMV and EC analyses. *p < 0.05 with FDR corrected.

Two-sample *t*-test results of effective connectivity to the left PG are illustrated in Figure 2 and Table 3. Results from the effective connectivity analysis showed significantly decreased causal inflow to the left PG from the frontal and limbic regions [including the left OFC, right calcarine, and bilateral supplementary motor area (SMA) in the obesity group] (*p* < 0.05, FDR corrected).

**TABLE 3:** Brain regions showing decreased effective connectivity to the left parahippocampal gyrus.

Region	MNI coordinates			Size (voxels)	Peak *t*-value
	
	x	y	z		
Left orbitofrontal cortex	−27	60	−3	23	−4.7981
Right calcarine	21	−63	9	26	−4.5161
Right supplementary motor area	10	3	48	24	−4.5273
Left supplementary motor area	−6	−12	51	23	−4.0273

*MNI, Montreal Neurological Institute.*

#### Effective Connectivity From the Left Parahippocampal Gyrus to the Whole Brain

**FIGURE 3:**
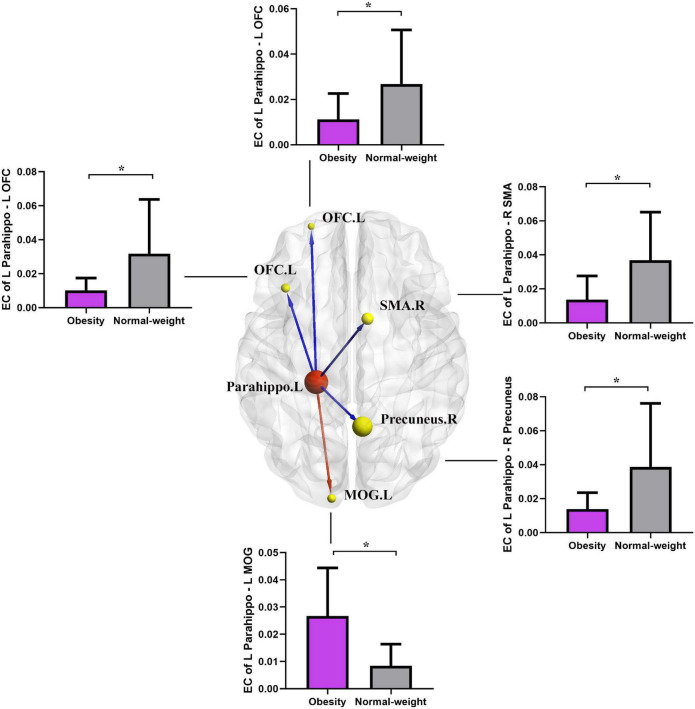
Altered EC in the obesity group compared with the normal-weight group (*p* < 0.05, FDR corrected). The red node indicates the key structural region (i.e., parahippocampal gyrus). The yellow nodes and blue lines indicate the output brain regions (SMA, OFC, and precuneus) with decreased EC output from the key region, and the red line indicates the increased EC output from the parahippocampal gyrus to the MOG. Parahippo, parahippocampal gyrus; SMA, supplementary motor area; OFC, orbitofrontal cortex; MOG, middle occipital gyrus; EC, effective connectivity; FDR, false discovery rate; GMV, gray matter volume; L, left; R, right. The size of the sphere represents the voxel size of the brain region based on the results of the GMV and EC analyses. *p < 0.05 with FDR corrected.

As shown in Figure 3 and Table 4, the two group comparisons revealed decreased causal outflow to the frontoparietal and limbic regions (including the left OFC, right precuneus, and right SMA) from the left PG and increased causal outflow to the left middle occipital gyrus (MOG) from the left PG in the obesity group compared with the normal-weight group (*p* < 0.05, FDR corrected).

**TABLE 4:** Brain regions showing decreased and increased effective connectivity from the left parahippocampal gyrus.

Region	MNI coordinates			Size (voxels)	Peak *t*-value
	
	x	y	z		
**Decreased**					
Left orbitofrontal cortex	−39	24	−3	24	−4.5441
	−24	60	−3	19	−4.4864
Right precuneus	6	−57	39	49	−4.2723
Right supplementary motor area	9	6	42	31	−4.0723
**Increased**					
Left middle occipital gyrus	−12	−99	0	23	4.1116

*MNI, Montreal Neurological Institute.*

### Correlation Analysis Between Altered Effective Connectivity and Dutch Eating Behavior Questionnaire Scores

**FIGURE 4:**
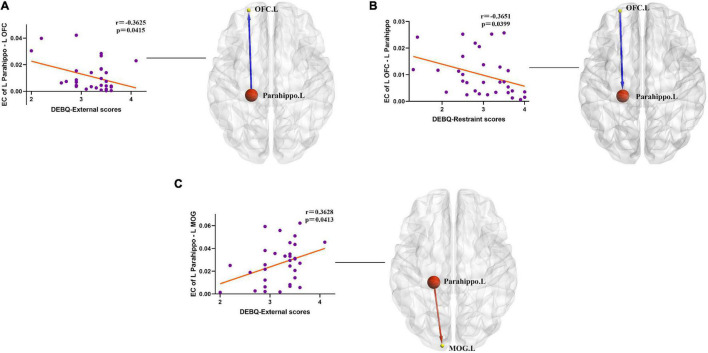
**(A)** Negative correlation between ΔDEBQ-External scores and causal outflow from the left parahippocampal gyrus to the left OFC (*r* = −0.3625, *p* = 0.0415). Parahippo, parahippocampal gyrus; OFC, orbitofrontal cortex; EC, effective connectivity; DEBQ, Dutch Eating Behavior Questionnaire; L, left; R, right. The size of the sphere represents the voxel size of the brain region based on the results of the GMV and EC analyses. **(B)** Negative correlation between ΔDEBQ-Restraint scores and causal inflow from the left OFC to the left parahippocampal gyrus (*r* = −0.3651, *p* = 0.0399). Parahippo, parahippocampal gyrus; OFC, orbitofrontal cortex; EC, effective connectivity; GMV, gray matter volume; DEBQ, Dutch Eating Behavior Questionnaire; L, left; R, right. The size of the sphere represents the voxel size of the brain region based on the results of the GMV and EC analyses. **(C)** Positive correlation between ΔDEBQ-External scores and causal outflow from the left parahippocampal gyrus to the left middle occipital gyrus (*r* = 0.3628, *p* = 0.0413). Parahippo, parahippocampal gyrus; MOG, middle occipital gyrus; EC, effective connectivity; DEBQ, Dutch Eating Behavior Questionnaire; L, left; R, right. The size of the sphere represents the voxel size of the brain region based on the results of the GMV and EC analyses.

In the obesity group, negative correlation was observed between DEBQ-External scores and causal outflow from the left PG to the left OFC (*r* = −0.3625, *p* = 0.0415) (Figure 4A). In addition, we found a negative correlation between DEBQ-Restraint scores and causal inflow from the left OFC to the left PG (*r* = −0.3651, *p* = 0.0399) (Figure 4B) and positive correlation between DEBQ-External scores and causal outflow from the left PG to the left MOG (*r* = 0.3628, *p* = 0.0413) (Figure 4C) in the obesity group. No additional significant correlations were found in the normal-weight group.

## Discussion

This study further explored the characteristics of brain reorganization of obesity. Specifically, at the structural level, the obesity group showed increased GMV alterations in the PG than the normal-weight group. At the EC level, the obesity group had significantly decreased interactions between the PG and OFC and PG and SMA. Additionally, decreased causal outflow from the PG to precuneus and causal inflow from the calcarine to PG were observed in this study. Increased causal outflow from the PG to the middle occipital gyrus was also found in the obesity group. The EC of PG–OFC was negatively associated with the ΔDEBQ-External/Restraint scores, and the EC of PG–MOG were positively associated with the ΔDEBQ-External score. Our findings may shed some light on the role of brain functional and structural alterations and their associations in obesity, and provide a better understanding of the neural mechanisms underlying the obesity.

Parahippocampal regions are part of the paralimbic cortex and have important roles, not only in memory and learning but also in the hedonic processing of feeding and incentive motivation (Bragulat et al., 2010). The evidence from previous studies has shown that the hippocampal function (i.e., hippocampus and PG) has been participating in the encoding of emotional memory and incentive salient stimuli memory, which contributes to associative learning and retrieval of food-related recollection (Berthoud, 2002; Bragulat et al., 2010). A series of neuroimaging studies further have found an increased activity of the PG in the context of food reward processing and state-like cravings, and this type of activation pattern was positively associated with obesity status and weight change (Haase et al., 2011; Brooks et al., 2013; Yokum et al., 2014). A task-based fMRI study has found that obese subjects had greater activation than normal-weight subjects in the hippocampus/parahippocampal area, while they are smelling food-related odors (Bragulat et al., 2010). Another longitudinal study revealed that percent weight loss in dieters with obesity was positively correlated with baseline GMV in PG (Honea et al., 2016). In addition, evidence from a task-based fMRI study has shown that significantly decreased activation of the PG was observed in individuals with severe obesity after bariatric surgery when they were exposed to high-energy dense vs. low-energy dense visual and auditory food cues, and their subjective cue-induced food craving correlated positively with brain activation in the PG (Baboumian et al., 2019; Bach et al., 2021). A previous study has examined the effect of the FTO rs9939609 genotype on the neural responses to visual food cues and found that the magnitude of the neural response to hedonic food images in FTO risk allele and non-risk genotype subjects at the fasted state was differentially modulated by fasted circulating acyl–ghrelin concentrations within the parahippocampus (Karra et al., 2013). Our previous findings further revealed that the PG, as a key functional node in the neural reward network of obesity, is directly involved in the cross-hemispheric reward processing pathway (Zhang et al., 2020, 2021). On the one hand, the results of this study show that the increased GMV in the PG supports the previous findings, which further suggest that the PG is not only associated with emotional and incentive mnemonic appetite drive, but may also be involved in the regulation of energy metabolism and is significantly associated with weight status. On the other hand, it is important to note that the results of the present study may be different from previous neuroimaging studies of obesity using similar study designs (García-García et al., 2019; Herrmann et al., 2019; Chen et al., 2020). This phenomenon may be caused by a combination of many confounding factors, but we believe that the obesity degree of enrolled volunteers may be an important part of it. The previous study have found that the neural plasticity changes in Class III obesity may be different from the individuals with Class I/II obesity (Nock et al., 2020).

The human brain is a complex network with some fundamental organizational principles (Bullmore and Sporns, 2009). As previous studies have proven, obesity is associated with a range of brain changes that include not only regional abnormalities but also impairments in the functional status of brain networks (Makaronidis and Batterham, 2018). In this study, decreased interaction between the paralimbic cortex (parahippocampal gyrus), a key structural node, and OFC was observed in the obesity group. Increasing pieces of evidence indicate that OFC neuronal activity encodes representations of outcomes and their subjective value in an identity-specific manner (Critchley and Rolls, 1996; Tremblay and Schultz, 1999; de Araujo et al., 2006; Jennings et al., 2019). The disruption of the OFC with obesity may result in altered value representations of food. A series of neuroimaging studies have reported that decreased OFC GMV, decreased total OFC volume, and altered fluid distribution in diffusion-weighted imaging were found in individuals with obesity or morbidly obesity (Alkan et al., 2008; Raji et al., 2010; Cazettes et al., 2011). In terms of functional imaging, task-based fMRI research has found increased activation of the OFC in individuals with obesity when they respond to visual food cues, and the activation pattern persisted even when they were sated, suggesting that the obesity group appears to be more responsive to food cues (Pursey et al., 2014). The OFC encodes general reward value and subjective value. Value encoding is the scaling of activation with the subjective value of the reward; this can be associated with food, monetary, or other rewards (Howard et al., 2015). Moreover, the OFC plays an important role in response inhibition in multiple tasks and disorders (Kringelbach and Rolls, 2004). Similar findings have been found in the field of task-based fMRI. Performance on the Stroop task was also correlated with BMI: participants with a higher BMI showed decreased response inhibition (Gunstad et al., 2007).

​​​​In a go/no-go task modified to include food cues, participants with obesity made more errors than normal-weight controls, and this finding was correlated with decreased activation of the OFC during this task in the obesity group (Batterink et al., 2010; Mobbs et al., 2011). Evidence from gene-related task-based fMRI study shows that people with the FTO risk allele genotype had increased activity in the OFC compared with the lower-risk genotype when they respond to “fattening” food image content (Melhorn et al., 2018). The role of OFC and paralimbic cortex (parahippocampal gyrus) in executive control and reward processing has been further supported by recent neuroimaging studies that found a decreased effective connection between the prefrontal cortex and the paralimbic cortex in people with internet gaming disorder (Wang et al., 2020). In our cohort, decreased interaction influence was observed between OFC and paralimbic cortex (parahippocampal gyrus), and this pattern of interaction was associated with restraint and external eating behavior scores. Based on previous findings, we speculate that emotional or memory-driven reward devalue processes may integrate deficits in executive control and amplification of external sensory signal processing in obesity to induce food or substance addiction.

The SMAs are involved in movement inhibition, response selection, and execution of behavior (Haggard, 2008; van der Laan et al., 2014). The SMA in the pathway from the basal ganglia to pre-SMA and SMA to the motor cortex has increasingly been proven to be involved in executive control and inhibition (Haggard, 2008). Growing evidence suggests that the (pre-)SMA activates during inhibitory actions to food and non-food stimuli (Hendrick et al., 2012; Pawliczek et al., 2013; Tuulari et al., 2015). Additionally, a previous study has demonstrated that emotional stimuli can influence how the SMA influences excitability of the motor cortex (Oliveri et al., 2003). Task-based fMRI studies have shown that more successful self-controllers showed increased activation in the SMA during high-energy snack food choices, which suggests a role of the SMA in food-related self-control (van der Laan et al., 2014). Our previous study also found that SMA plays an important role in planning and execution of behavior and directly participates in the regulation loop of key reward nodes (Zhang et al., 2021). Previously using VBM, other researchers also observed that a lack of self-control and self-inhibition was related to lower GMV in the SMA (Matsui et al., 2002). For eating behavior, a neuroimaging study found that lower GMV in the SMA might be related to self-reported restrained eaters’ reduced inhibitory capacity (van der Laan et al., 2016). In this study, decreased interaction influence between paralimbic cortex (parahippocampal gyrus) and SMA in the present study may reflect the impaired integration interoceptive signals of the reward devaluation mediated by emotional awareness and behavior inhibition in obesity.

Extant manuscript reported that a part of the middle occipital gyrus and calcarine cortex (lower visual areas) are involved in visual processing and sustained attention (Lalli et al., 2006; Murdaugh et al., 2012). Research has suggested that brain regions known to mediate attention selectively to external stimuli are important for actively directing attention internally (Critchley et al., 2004; Nebel et al., 2005). Behavioral studies of attention have found that hungry individuals with obesity have increased directed attention to food images or faster reaction times to food images, and after viewing such food images, show increased food intake compared with hungry normal-weight controls (Nijs et al., 2010; Yokum et al., 2011). In addition, more evidence has shown that individuals with obesity continued to have sustained increased attention to food images, even when they were sated (Castellanos et al., 2009). Based on previous findings, scholars propose a neurofunctional-based addiction model, which is thought to be composed of dysfunction in self-control and attentional bias to external stimuli, to explain why some individuals with obesity have difficulty regulating caloric intake and maintaining energy homeostasis (Volkow et al., 2013). This study expands on previous research suggesting that disturbances in the integration of signals between the paralimbic cortex and visual and attentional functional areas may reflect the disengagement of attentional and visual processes for external sensory signals, which may increase vulnerability to food-related addictive behaviors or obesity, and reduce the neural threshold for external eating behaviors.

## Limitations

It is important to note some of the limitations of this study. First, we were not able to exclude the other factors that would affect the whole-brain activity, such as metabolic hormone levels or different types of obesity, and menstrual phase has been shown to affect neural reward-associated activation (Dreher et al., 2007). In addition, we relied solely on BMI to assess obesity, although this is the standard recommended by the WHO, but it lacks some objective indicators, such as body fat percentage and waist-to-hip ratio, to make a comprehensive assessment of obesity. Due to the limited sample size, we did not compare the grouping of obesity studies based on different classes, which may obscure some meaningful results. Finally, we studied individuals in a fasted state only, and it would be interesting in future studies to compare results obtained with different class obesity individuals in both a fasted and sated state, and combined with specific objective indicators, eating habits, and living habits.

## Conclusion

In summary, individuals with obesity show different patterns of neural structure and connectivity in the brain regions. First, the paralimbic cortex (parahippocampal gyrus) was identified as a key neural structural brain region in obesity. Moreover, the alterations in key neural structure may be caused by hierarchical functional integrated alterations such as devalued reward system driven by emotion and incentive, disordered behavioral inhibition, and the disengagement of attentional and visual function for external sensory signals. Given the significant and increasing prevalence of obesity, these findings have important implications for understanding neural mechanism in obesity, particularly in individuals with abnormal eating behaviors. We hope the results will help future research in developing individual-tailored strategies for obesity prevention.

## Data Availability Statement

The raw data supporting the conclusions of this article will be made available by the authors, without undue reservation.

## Ethics Statement

The experimental protocol was approved by the Institutional Review Board of Beijing Friendship Hospital. The study was conducted in accordance with the Declaration of Helsinki. All participants were informed of the nature of the research. The patients/participants provided their written informed consent to participate in this study.

## Author Contributions

PZ was responsible for the study conception, the data collection, the analysis and interpretation, drafting, and critical revision of the manuscript. G-WW, F-XY, and YL were responsible for the statistical analysis, interpretation of the data, and critical revision of the manuscript. L-RT, Z-TZ, M-YL, Z-HY, and YL were responsible for the data collection, interpretation of the data, and revision of the manuscript. ZW, HL, and Z-CW were responsible for the interpretation of the data and critical revision of the manuscript. HL, YL, and Z-CW were the corresponding authors of this manuscript and agreed to be accountable for all aspects of the work. All authors contributed to the article and approved the submitted version.

## Conflict of Interest

The authors declare that the research was conducted in the absence of any commercial or financial relationships that could be construed as a potential conflict of interest.

## Publisher’s Note

All claims expressed in this article are solely those of the authors and do not necessarily represent those of their affiliated organizations, or those of the publisher, the editors and the reviewers. Any product that may be evaluated in this article, or claim that may be made by its manufacturer, is not guaranteed or endorsed by the publisher.

## Footnotes

1
http://www.neuro.uni-jena.de

2 https://www.fil.ion.ucl.ac.uk/spm, version 12

3
http://rfmri.org/dpabi

4
https://www.nitrc.org/projects/gretna/

5
www.restfmri.net/forum/DynamicBC
